# Impact of repeated ivermectin treatments against onchocerciasis on the transmission of loiasis: an entomologic evaluation in central Cameroon

**DOI:** 10.1186/1756-3305-6-283

**Published:** 2013-09-27

**Authors:** Marc K Kouam, Jules B Tchatchueng-Mbougua, Maurice Demanou, Michel Boussinesq, Sébastien DS Pion, Joseph Kamgno

**Affiliations:** 1 Filariases and other Tropical Diseases Research Center (CRFilMT), P.O. Box 5797, Yaoundé, Cameroon; 2 Department of Animal Production, University of Dschang, P.O. Box 222, Dschang, Cameroon; 3 Centre Pasteur of Cameroon, P.O. Box 1274, Yaoundé, Cameroon; 4 Institut de Recherche pour le Développement (IRD), P.O. Box 64501, Montpellier, France; 5 Université de Montpellier 1, 5 Boulevard IV, Montpellier 34000, France; 6 Faculty of Medicine and Biomedical Sciences University of Yaoundé I, P.O. Box 1364, Yaoundé, Cameroon

**Keywords:** CDTI, Entomologic indices, *C. silacea*, *C. dimidiata*, Loiasis, Cameroon

## Abstract

**Background:**

Annual community-directed treatment with ivermectin (CDTI) have been carried out since 1999 in the Lekie division (central region of Cameroon where most cases of *Loa*-related post ivermectin severe adverse events were reported) as part of the joined activities of the African Programme for Onchocerciasis Control (APOC) and Mectizan® Donation Program (MDP). As large-scale administration of ivermetine was demonstrated to be an efficient means to control loiasis transmission, it was hypothesized that CDTI would have lowered or halted the transmission of *Loa loa* in the Lekie division after 13 years of annual drug administration, indicating a possible reduction in the occurrence of *Loa*-related post-ivermectin severe adverse events.

**Methods:**

A 4-month entomologic study was carried out from March to June 2012 in the Lekie division to evaluate the impact of 13 years of CDTI on the transmission of *L. loa* whose baseline data were recorded in 1999–2000.

**Results:**

There was a significant reduction in the infection rate for *Chrysops silacea* and *C. dimidiata* from 6.8 and 9% in 1999–2000 to 3 and 3.6% in 2012, respectively. The differences in the infective rate (IR) (percentage of flies harboring head L3 larvae), potential infective rate (PIR) (percentage of flies bearing L3 larvae), mean head L3 larvae load (MHL3) (average L3 per infective fly) and mean fly L3 larvae load (MFL3) (average L3 per potentially infective fly) for both *C. silacea* and *C. dimidiata* were not significantly different between the two investigation periods. The biting density (BD) was almost three-fold higher in 2012 for *C. silacea* but not for *C. dimidiata*. The transmission potential (TP) which is a function of the BD, was higher in the present study than in the baseline investigation for each species.

**Conclusion:**

The infection rate remaining high, the high TP and the stability observed in the IR, PIR, MHL3 and MFL3 after 13 years of CDTI suggest that transmission of *L. loa* is still active. This is an indication that the risk of occurrence of severe adverse events such as fatal encephalopathies is still present, especially for heavily microfilaria-loaded people taken ivermectin for the first time.

## Background

Control programs against onchocerciasis and lymphatic filariasis are based on mass treatment with ivermectin (IVM) and a combination of IVM and albendazole, respectively. Since 1991, serious adverse events (SAEs), including fatal cases of encephalopathy have been recorded in areas where onchocerciasis and loiasis (*Loa loa* filariasis) are co-endemic. In the following years, it has been shown that these accidents occur in individuals presenting a *L. loa* microfilaremia exceeding 30,000 microfilariae per milliliter of blood (mf/ml) [1-4]. *L. loa* is transmitted by tabanids belonging to the genus *Chrysops* and is highly endemic in forested regions as well as in some savanna areas of west and central Africa [5,6]. In 2000, the African Programme for Onchocerciasis Control (APOC) and the Mectizan® Donation Program (MDP) set up a specific surveillance system in areas where onchocerciasis and loiasis are co-endemic, for an early detection and management of SAEs [7,8].

This strategy has been successful in terms of reduction of lethality rate, but is somewhat costly to put in place [9]. Thus, the issue has been raised as to whether it should be maintained for as long as the community-directed treatment with IVM (CDTI) continues. Indeed, as IVM brings about a long-lasting decrease in *Loa* microfilaremia [10,11], almost all those individuals who have received the drug at least once are no longer at risk of developing a SAE. This is supported by the fact that more than 90% of the SAEs occur after a first treatment with IVM [12]. During the subsequent CDTIs, the surveillance system has to be maintained to detect and manage SAEs only in those adults who have never taken the drug for various reasons (refusals, migrants from untreated areas, repeated contra-indications) and in all children reaching the age of 5 for whom IVM was formerly contra-indicated. Maintaining the system is justified by the fact that during the first rounds of CDTI, the situation of these untreated persons regarding their infection with *L. loa* remains probably unchanged. However, on a longer term, this is probably not the case because the decrease in the microfilarial reservoir following mass IVM treatment might also reduce the number of parasites ingested and retransmitted by the vectors, and thus have a beneficial indirect effect on the total population, including those who have never taken the drug. Actually, this reduction in the transmission of *L. loa* in the CDTI area would have two effects. First, in uninfected or little infected children the reduction in the number of incoming infective larvae (L3) would have an impact on the number of adult worms developing, which might prevent them to present a high microfilaremia. Second, in adult subjects who already harbour adult worms, and do not receive the drug, it would lead to a slow reduction in the microfilaremia due to the fact that the number of worms dying naturally would exceed the number of new parasites. However, in the latter case, the time after which this effect would occur is not known because one lacks information on the mean lifespan of adult *L. loa* - the only information available is on its maximum, which could be 20 years [13].

The objective of the present study was to evaluate the impact of repeated annual IVM treatment on the intensity of transmission of loiasis, and thus on the possible reduction in the risk of SAEs in the untreated population. Such an evaluation can be made by two means: either by comparing the microfilarial loads found in untreated children before and after the implementation of the program, as it has been done for onchocerciasis [14], or by comparing the intensity of infection in the *Chrysops* vectors. Such an entomologic approach has been used to evaluate the impact of three monthly treatments on *Loa* transmission [15], but never that of annual CDTI. Consequently, an entomologic study was conducted in a village of the Lekie division (a division of the central region of Cameroon where most of the cases of SAEs recorded in the country occurred) where the transmission of loiasis had been characterized in detail in 1999–2000 at the early beginning of CDTI.

## Methods

### Study area

**Figure 1:**
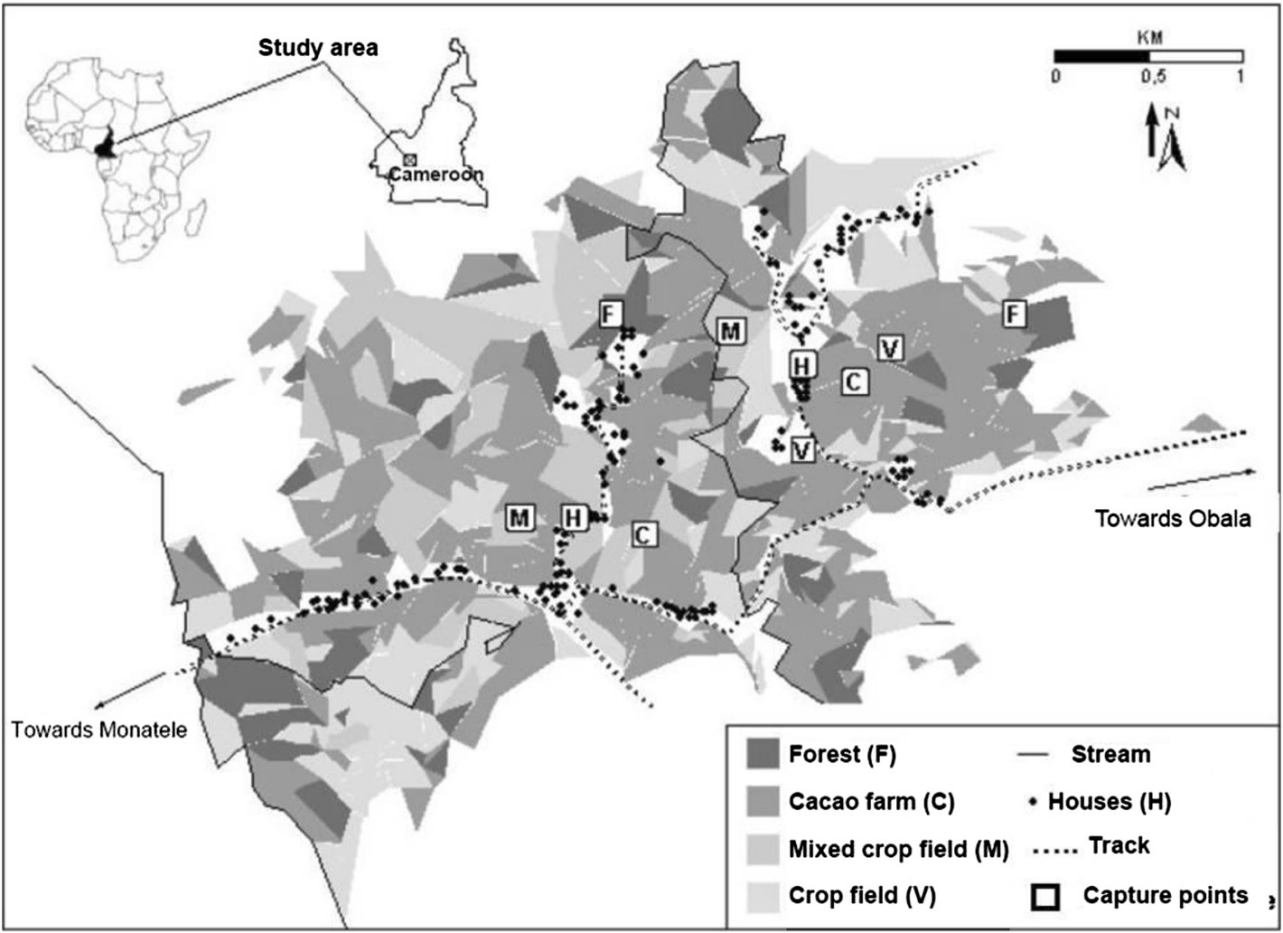
**Map of Kokodo, with location of capture points (adapted from **[16]**, with author’s permission).**

This site (Figure 1) was already described in detail by Demanou *et al*. [16]. Brievely, Kokodo (4°12’N, 11°18’E) is located in the Elig Mfomo subdivision, about 70 km north-west of Yaounde, in an area of semi-degraded forest. Elig Mfomo is within the Lekie division where most cases of SAEs have been documented [4,16]. The climate is equatorial with four seasons comprising two rainy seasons from September to mid-November, and from mid-March to June. The annual rainfall ranges between 1600 and 2000 mm, and the mean annual temperature is 25°C. In 2010, the total population in Kokodo was 1615 persons.

No survey on onchocerciasis has ever been conducted in Kokodo. However, the parasitologic surveys and rapid epidemiologic assessments (based on the prevalence of nodules) performed in the 1990s in neighboring villages, have shown that the area is at least mesoendemic for onchocerciasis (Boussinesq, unpublished data).

### Previous studies on loiasis in Kokodo

In January 1996, the village was included in a study aiming at identifying the risk factors associated with post-IVM SAEs [4]. Detailed data on Kokodo currently presented, though part of the study by Gardon *et al*. [4] in the Lekie division, were not previously published. A total of 453 persons aged 15 years and above participated in the study. All individuals were first asked whether they had already received IVM treatment, and 107 (23.6%) declared they had so (3 in 1993, 72 in 1994 and 32 in 1995). This relatively high proportion of persons already treated was due to the fact that large-scale IVM treatments were organized since 1993 by the Ministry of Public Health and the non-governmental development organization (NGDO) Helen Keller International (HKI) in the neighboring sub-division of Monatele. After having been registered, all those persons aged 15 years and above and who had never taken IVM (N = 69) were invited to provide blood by fingerprick to prepare blood smears to assess the prevalence and intensity of *Loa* microfilaremia. All the 453 volunteers received a single dose of IVM (150 μg/kg) just after sampling. Subsequent examination of the blood smears showed that the prevalence of *Loa* microfilaremia in the 69 ivermectin-naïve adults was 29.0% and the arithmetic mean microfilarial density among the microfilaremics was 10,401 mf/ml.

In 1999, a second study was implemented in Kokodo whose objectives were (i) to characterize the transmission of *L. loa* by examining *Chrysops* caught over 12 months (May 1999-April 2000) in various places of the village [16] and (ii) to identify individual factors associated with the presence of *Loa* microfilaremia [17]. For the second part of the study, all residents aged ≥ 15 years were again invited to be examined for *Loa* infection in April 1999, just before the first CDTI campaign launched by APOC in the area. Among the 441 who volunteered, 141 (32.0%) declared they had received IVM after the 1996 study. The prevalence of *Loa* microfilaremia among the adults aged ≥15 years who had not received IVM between 1996 and 1999 was 29.0% (like in 1996) and the arithmetic mean microfilarial density among the microfilaremics was 8861 mf/ml.

### Ivermectin treatment in Kokodo

As mentioned above, Kokodo is not far from the border with the Monatele sub-division where large-scale IVM treatments have been organized from 1993 to 1998 by the Ministry of Public Health and HKI. Thus, a number of persons living in Kokodo could receive treatment during this period. Since 1999, Kokodo has been treated annually under the auspices of APOC, following the CDTI strategy. Drug coverages in the village itself are unknown for the years 1999–2006 but figures on the whole CDTI project (“Centre 3”) were available for this period. For the years 2007–2010, coverage data on the village of Kokodo could be provided by the National Program for Onchocerciasis Control and HKI.

### Catches and dissection of *Chrysops* in 2012

Catches were organized from 1 March to 30 June 2012, in exactly the same sites and conditions as in the first entomologic study [16]. This four-month period was chosen because it started and ended 6 and 10 months respectively, after the end of the previous CDTI campaign (August 2011). The captures were conducted in 10 catching stations located in 5 types of environment (forest, cacao farms, crop fields, mixed crop fields and inhabited areas). Two catching stations were chosen for each of these types, and catches of *Chrysops* were made every two weeks during three consecutive days. In this paper, data for all habitats were pooled for each study period. Flies were caught using hand nets from 07:00 to 18:00 by collectors stationed by a wood fire, the smoke of which attracts the flies [18,19]. One collector was allowed to catch between 7:00 and 12:00 and another one took over from 12:00 to 18:00. Each collector was rotated to a different caching station every day. The flies caught were put singly in a glass tube plugged with a cotton wool and grouped in bags labeled according to the catching station, environment type and hour. Bags were collected every two hours and brought back to the field laboratory where flies were first identified, then dissected alive after a slight knock down using a needle tip, and examined for parity and presence of *L. loa* larvae (first stage L1, second stage L2, and third stage L3 larvae). Dissection was carried out by a single individual. Identification of larvae was performed following the method of Orihel and Lowrie [20].

The head, thorax and abdomen were dissected separately. The infection rate was defined as the proportion of flies containing any larval stage (L1, L2 or L3), and the infective rate (IR) as the proportion of flies with L3s in the head or mouth parts. The potential infective rate (PIR) was considered as the proportion of dissected flies with L3 larvae, irrespective of their location in the fly. The infection and infective rates have been calculated using, first, the total number of flies, and then the number of parous flies, as the denominator. The mean head L3 load (MHL3) corresponded to the total number of L3s found in the head and mouth parts divided by the total number of *Chrysops* carrying L3s in this location. The mean fly L3 load (MFL3) was determined as the total number of flies with L3 larvae divided by the total number of flies with these larvae. The monthly transmission potential (MTP) was expressed as the number of infective larvae per man per month (L3/man/month), while the monthly biting density (MBD) was estimated as the mean number of fly bites per person per month (bites/man/month). The calculations of MBD and MTP were made using the formula used as part of studies on onchocerciasis transmission [21].

### Statistical analyses

As mentioned above, the first entomologic study was conducted over 12 months, i.e. between 1 May 1999 and 30 April 2000. As the indicators of interest in this study (infection rate, IR, PIR, MHL3 and MFL3) fluctuate seasonally [16,22-24], we decided to compare the figures obtained in 2012 with those obtained during the same months in 1999 and 2000 (March and April 2000, and May and June 1999). The non parametric Wilcoxon rank two-sample test was used to compare the MHL3 and MFL3 between 1999–2000 and 2012. Chi-square test was used to compare the infection rates, IR and PIR between the two periods. These comparisons were done on the infection and infective rates in the total number of *Chrysops*, and then on the rates in the parous flies only. Fischer’s exact test was used in place of Chi-square test whenever the expected value was less than 5. The significance level was set at 0.05 in all cases. All statistical analyses were performed using the STATA 12.1 software (STATA Corporation, College Station, TX, USA).

## Results

### Entomologic indices

During the initial study, the total numbers of *Chrysops* caught during the 4 month-period (March and April 2000, May and June 1999) was 982, and 976 of them were dissected (Table 1). *C. silacea* accounted for 85.2% of catches (14.8% for *C. dimidiata*). The infection and infective rates did not differ significantly between both species (p = 0.94 and 0.89, respectively). The MHL3 was higher for *C. silacea* than for *C. dimidiata* but due to the low number of infective *C. dimidiata*, the significance in the difference could not be computed. Of the 976 flies dissected, 27 (2.8%) carried L3s (23 *C. silacea* and 4 *C. dimidiata*). The PIR was 2.8% for each of the two species. The MFL3 was 123 L3/fly for both species combined, and 141 and 18 L3/fly for *C. silacea* and *C. dimidiata* respectively.

**Table 1:** Entomologic indices at baseline study (1999–2000)

**Study period**		No. of *chrysops *caught*			**BD**			**TP**			**Infection rate (%)**			**Infective rate (%)**			**MHL3 load**		
		**S**	**D**	**S+D**	**S**	**D**	**S+D**	**S**	**D**	**S+D**	**S**	**D**	**S+D**	**S**	**D**	**S+D**	**S**	**D**	**S+D**
**March**		63	10	73	32.6	5.2	37.8	55.6	0.0	55.6	3.6	20.0	6.2	3.6	0.0	3.1	53.5	0.0	53.5
**April**		251	56	307	125.5	28.0	153.5	754.9	9.0	763.9	9.3	12.5	9.9	3.6	3.6	3.6	162.5	9.0	134.5
**May**		184	41	225	47.5	10.6	58.1	12.4	0.0	12.4	7.0	0.0	5.8	1.6	0.0	1.3	16.0	0.0	16.0
**June**		339	38	377	78.2	8.8	87.0	95.1	4.6	99.7	5.3	10.5	5.8	0.9	2.6	1.1	137.4	20.0	108
	**Total**	*837*	*145*	*982*	*283.8*	*52.6*	*336.4*	*918*	*13.6*	*931.6*	*6.7*	*9.0*	*7.1*	*2.1*	*2.1*	*2.1*	*119.4*	*12.7*	*103.4*
	**p-value**	-		-		-		-			0.94		-	0.89			-		

*All the *Chrysops* were dissected except six *C. silacea* caught in March (2) and April (4); BD= biting densities; TP= transmission potential; MHL3= mean head L3 load; S= *Chrysops silacea*; D= *Chrysops dimidiata*; S+D= both species combined.

Between March and June 2012, a total of 1559 *Chrysops* was caught and 1558 were dissected (Table 2). As previously observed in the baseline study, *C. silacea* was found to predominate, with a proportion of 92.9% of the *Chrysops* caught. Like in 1999–2000, the infection and infective rates were quite similar between both species (p = 0.94 and 0.89, respectively) and the MHL3 was higher in *C. silacea* than in *C. dimidiata* (again, the significance of the difference could not be computed due to the low number of infective *C. dimidiata*)*.* Of the 1558 flies dissected, 33 (2%) carried L3s (30 *C. silacea* and 3 *C. dimidiata*). The PIR were 2.0% and 2.8% for *C. silacea* and *C. dimidiata* respectively. The MFL3 were 122 and 121 L3/fly for *C. silacea* and *C. dimidiata* respectively, and 121 L3/fly for both species combined.

**Table 2:** Entomologic indices in the present investigation (2012)

**Study period**		No of *chrysops *caught*			**BD**			**TP**			**Infection rate (%)**			**Infective rate (%)**			**MHL3 load**		
		**S**	**D**	**S+D**	**S**	**D**	**S+D**	**S**	**D**	**S+D**	**S**	**D**	**S+D**	**S**	**D**	**S+D**	**S**	**D**	**S+D**
**March**		282	42	324	213.9	39.3	253.2	572.5	0.0	572.5	3.9	2.4	3.7	2.5	0.0	2.2	100.7	0.0	100.7
**April**		270	31	301	136.0	17.0	153.0	204.6	0.0	204.6	3.3	0.0	3.0	2.2	0.0	2.0	67.7	0.0	67.7
**May**		584	26	610	303.8	15.5	319.3	30.2	17.3	47.5	2.1	11.5	2.5	0.9	11.5	1.3	11.6	9.7	10.9
**June**		313	11	324	157.0	7.0	164.0	352.0	0.0	352.0	3.5	0.0	3.4	1.9	0.0	1.9	117.3	0.0	117.3
	**Total**	*1449*	*110*	*1559*	*810.5*	*78.8*	*889.5*	*1159.3*	*17.3*	*1176.6*	*3.0*	*3.6*	*3.0*	*1.7*	*2.7*	*1.7*	*78.0*	*9.7*	*70.0*
	**p-value**	**-**			**-**			**-**			0.94			0.89			**-**		

*All the *Chrysops* were dissected except one *C. silacea* caught in March; BD= biting densities; TP= transmission potential; MHL3= mean head L3 load. S= *Chrysops silacea*; D= *Chrysops dimidiata*; S+D= both species combined.

### Comparison of the entomologic indicators recorded in 1999–2000 and in 2012

Results of comparisons are presented in Table 3. In the initial study, the infection and infective rates of *C. silacea* during the four months were 6.7 and 2.1%, respectively, whereas in 2012, the same indicators for this species were 3.0% and 1.7% respectively. The infection rate for this species was significantly lower in 2012 than in 1999–2000 (p = 0.001). In contrast, the infective rates recorded during the two periods for this species did not differ significantly (p = 0.49). Similarly, the infection rate was significantly lower for *C. dimidiata* in 2012 than in the baseline study (p = 0.04) while no significant difference in the IR for this species was reported between the two periods of investigation (p = 0.73). Considering both species pooled together, the infection rate in 2012 (3.0%) was significantly lower than in the baseline study (7.1%) (p = 0.001), while the infective rate (1.7% in 2012 and 2.1% in initial study) was not (p = 0.55).

**Table 3:** Comparison of entomological indices between the two study periods (baseline study (1999–2000) and present investigation)

**Study period**		**Infection rate (%)**			**Infective rate (%)**			**Potential infective rate**			**MHL3 load**			**MFL3 load**		
		**S**	**D**	**S+ D**	**S**	**D**	**S+ D**	**S**	**D**	**S+D**	**S**	**D**	**S+ D**	**S**	**D**	**S+D**
**Baseline**		6.7	9.0	7.1	2.1	2.1	2.1	2.8	2.8	2.8	119.0	13.0	103.0	141.0	18.0	123.0
**2012**		3.0	3.6	3.0	1.7	2.7	1.7	2.0	2.8	2.0	78.0	10.0	70.0	122.0	121.0	121.0
	**p-value**	*0.001*	*0.04*	*0.001*	0.49	0.73	0.55	0.29	0.99	0.30	0.91	0.51	0.91	0.84	0.48	0.55

NB: S= *Chrysops silacea*; D= *Chrysops dimidiata;* S+D= both species combined; MHL3= mean head L3 load; MFL3= mean fly L3 load.

The PIRs recorded in the 2012 study were not significantly different from that recorded in 1999–2000 and this holds true either for *C. silacea* alone, or *C dimidiata* alone, or both species combined (p = 0.29, 0.99 and 0.30 respectively).

There was no significant difference in the MHL3 between the two investigation periods for *C. silacea* (p = 0.91), *C. dimidiata* (p = 0.51), and both species combined (p = 0.91). Similarly, the difference in the MFL3 between the two studies was not significant for *C. silacea* (p = 0.84), *C. dimidiata* (p = 0.48) or both species pooled together (p = 0.55).

The biting density was almost three-fold higher in 2012 than in 1999–2000 for *C. silacea* but remained stable for *C. dimidiata*.

The transmission potential was higher in the present study than in the baseline investigation for each species or both species combined. There was a monthly variation in the transmission potential during both investigation periods (Tables 1 and 2).

The parous rate (Table 4) was significantly higher in the initial survey than in the second for *C. silacea* (p = 0.00007) and both species combined (p = 0.00001). As in the total *Chrysops* population dissected, there was a significant decrease in the infection rate for *C. silacea* and both species combined in parous fly population, from 19.4 and 20.1% respectively in the initial study to 11.1% in the present study (p = 0.002 and 0.0005 respectively). Also, the infective and potential infective rates between the two periods for each species and for both species combined were not significantly different in parous flies.

**Table 4:** **Comparison of infection, infective and potential infective rates in parous *Chrysops *between the two study periods**

**Study period**		**No of *chrysops *dissected**			**Number parous**			**Percentage parous (%)**			**Infection rate in parous (%)**			**Infective rate in parous (%)**			**Potential infective rate in parous**		
		**S**	**D**	**S+D**	**S**	**D**	**S+D**	**S**	**D**	**S+D**	**S**	**D**	**S+D**	**S**	**D**	**S+D**	**S**	**D**	**S+D**
**Baseline**		831	145	976	289	55	344	34.5	37.9	35.2	19.4	23.1	20.1	5.9	5.5	5.8	8	7.3	7.8
**2012**		1448	110	1558	389	34	423	26.9	30.9	27.2	11.1	11.8	11.1	6.2	8.8	6.4	7.7	8.8	7.8
	**p-value**							*0.00007*	0.24	*0.00001*	*0.002*	0.16	*0.0005*	0.87	0.53	0.74	0.90	0.79	0.98

NB: S= *Chrysops silacea*; D= *Chrysops dimidiata;* S+D= both species combined.

### Drug coverage

The drug coverages in the whole CDTI project area were 46% in 1999, 29% in 2000, 29% in 2001, 50% in 2002, 62% in 2003, 67% in 2004, 64% in 2005, and 79% in 2006. For the following years, data was available at the village level and the therapeutic coverage in Kokodo ranged from 67.9% in 2007 to 80.2% in 2010, with values of 79.5% and 79.9% in 2008 and 2009, respectively. Data related to 2011 were not available at the time of study.

## Discussion

As in the initial study by Demanou and others [16], *C. silacea* and *C. dimidiata* were the only species caught during the investigation period, *C. silacea* being the predominating species. Previous studies showed that *C. silacea* was the dominating species in all *Loa* endemic areas in Cameroon except in the village of Ngat, 50 km south of Yaounde where *C. dimidiata* is the main vector of loiasis [15]. Our study confirmed that *C. silacea* is the most important vector in the Lekie area, both because of higher MHL3 (7.5- fold higher than in *C. dimidiata*), and of higher biting densities (10.3- fold higher than *C. dimidiata* biting densities).

IVM is an effective drug against *O. volvulus, L. loa, Wuchereria bancrofti* and is currently recommended and used by different global programs for the control of onchocerciasis and lymphatic filariasis. Regarding loiasis specifically, several studies showed that a single dose of IVM brings about a marked and long-standing decrease in *Loa* microfilaremia, lowers the prevalence of loiasis [10,11], and reduces the infective rate of the *Chrysops* to low levels when given on a large scale at 3-monthly intervals [15]. Since large-scale administration of IVM was demonstrated to be an efficient means to control *Loa* transmission, it was hypothesized that CDTI would have had such a result in the Lekie division after several years of annual distributions. At the launching of CDTI in this division, entomologic data on the transmission of loiasis were recorded over 1 year, and distribution of IVM has been conducted annually, every year, with various therapeutic coverage rates. To evaluate the impact of this drug coverage on the transmission of loiasis, entomologic data were collected over a 4-month period from March to June 2012 and compared with the baseline data.

In 2012, the biting densities for *C. silacea* and *C. dimidiata* were 811 and 79 bites/man respectively for a 4- month study period, while for the same months and study duration in 1999–2000, these values were almost three-fold lower for *C. silacea* and nearly similar for *C. dimidiata*. Both species were shown to be sensitive to rainfall, but with a dominion of *C. silacea* over *C. dimidiata* in the rainy season [23]. The increase in the density of *C. silacea* but not *C. dimidiata* might be related to an increase in the rainfall in this area.

In the present study, we have used a number of indicators to evaluate the impact of CDTI on *Loa* transmission. As the infective forms of *L. loa* were suspected to be able to pass freely and fast from the head to the abdomen or vice-versa when the fly is feeding [25], flies carrying L3s were considered both when the latter were located in their head, or in other locations (thorax or abdomen). In spite of the significant reduction in *Loa* infection rate for each species (*C. silacea* and *C. dimidiata*) observed in 2012, the values of the IR, PIR, MHL3 and MFL3 recorded in 2012 were not significantly different from the initial values obtained in the baseline investigation for each species. The results were similar using both the total number of flies and the number of parous flies as the denominator. The stability in the values of these indices indicates that the level of exposure of *L. loa* has not changed after 13 years of treatment. Moreover, the intensity of infection remained high despite the significant drop observed in the infection rate. These surprising and disappointing observations could be attributed to several facts, some of which are: 1) the persistence of a significant parasite reservoir; 2) an increase in the density of vector flies; 3) the biting habits of the vector flies.

The parasite reservoirs are the microfilaria carriers, including the fraction of the population that remains untreated [26]. Among these individuals, some refuse to be treated by fear of SAEs. In this respect, it is worth noticing that in the past, the Lekie division had experienced many cases of SAEs [27]. In spite of the proper sensitization of populations through explanations of the advantages of taking IVM, reassurance about efficacy and safety about availability in health facilities of kits to manage possible SAE cases, some people are still reluctant to comply with the program. Other individuals who can act as reservoir are infected immigrants or former residents returning to the village from towns or areas where there is not treatment. A third group consists of residents remaining untreated due to shortage of drug during the CDTI campaign. Indeed, complaints on this issue were recorded by our team more than once in the field during *Chrysops* collection.

A second reason which may partly explain the limited impact of CDTI on entomologic indicators on *Loa* transmission is the increase in the density of *Chrysops* between 2000 and 2012. Whatever the reason(s) for this increase, this phenomenon increases the likelihood of a vector to bite an infected person and to pass on subsequently the infection to others, including healthy individuals.

Lastly, the biting behavior of *Chrysops* known as pool-feeding is very painful to the host. As a result, the fly usually bites multiple hosts in order to feed to repletion. This habit of multiplying the blood source increases the probability for the fly to feed on microfilaria carriers.

The fact that the infection rate was significantly reduced while the IR, PIR, MHL3 and MFL3 were not may be related to the reduction of parasite reservoir among the treated population for the first case, and to a phenomenon of “limitation” (where the parasite ‘success rate’ or ‘yield’ in the vector increases as the mf intake decreases) for the latter case. The parasite yield is high with *Chrysops*, as observed by Kershaw and Duke [28], and this might be due to the ability of *Chrysops* to live normally even when they harbor very high numbers of L3 [28,29]. However, there is little information on the type of relationships (limitation, facilitation or proportionality) between the number of *Loa* mf ingested by the *Chrysops* and the number of infective larvae. This is due to the fact that, the *Chrysops* bites being painful, it is difficult to infect experimentally these vectors by making them feed on humans. However, Kershaw and Duke [28] found that “the number of infective forms found in flies which survive long enough to support the development of the infective forms is similar to the number of microfilariae taken in”, suggesting the relationship is proportional. This does not explain the results obtained during the present study and should encourage conducting studies on this issue. *Chrysops* could be fed with blood from naturally infected human or from experimentally-infected baboons; in the latter case, blood meal could be taken directly on the host but in both cases, feeding could be made using a parafilm membrane technique [30].

In addition to these main facts, the therapeutic coverage as well as the duration of the treatment campaign within a year needs to be addressed. The official drug coverage from 2007 to 2010 ranged between 67.9 to 80.2%. With such fairly high coverages, one might have expected a significant reduction in the transmission level of loiasis after more than a decade of drug administration, not a standstill [31]. Therefore, another explanation to the stability in the indicators might be that the therapeutic coverage reported might be overrated. External assessment of therapeutic coverage should be encouraged to validate or revise official figures. Since onchocerciasis and loiasis are co-endemic in the study area, and the parasite reservoir (human population) is the same for both *O. volvulus* and *L. loa*, the stability in the transmission level of loiasis despite several years of CDTI might also be found for onchocerciasis. This is supported by the results of recent epidemiological investigations on onchocerciasis in the neighboring health district Mbam valley, which showed that some villages still had very high prevalences of *O. volvulus* infection (>50%) in spite of 15 rounds of annual CDTI (Boussinesq *et al*., unpublished data). The authorities in charge of onchocerciasis control program should consider the results of the present investigation for the better management of their activities in that area. Also, epidemiological studies on *L. loa* transmission in the Lekie division are necessary to complement this work.

## Conclusion

In conclusion, this study showed that the transmission of loiasis in the Lekie division is still active, as suggested by the infection rate remaining high, the high TP and the stability observed in the IR, PIR, MHL3 and MFL3 after 13 years of CDTI. This is an indication that the risk of occurrence of severe adverse events such as fatal encephalopathies is still present, especially for heavily microfilaria-loaded people taken ivermectin for the first time. Complementary data on the level of *Loa* microfilaremia are needed in the area.

## Competing interests

The authors declare that they have no competing interests**.**

## Authors’ contributions

All the authors have contributed significantly to this study. MKK, MB and JK contributed intellectually to the conceptualization and design of the study. JB T-M performed the statistical analysis. MKK and MD carried out the laboratories and field studies. MKK, MB, SDP and JK prepared the manuscript. All authors read and approved the final manuscript.
